# Radiomics models of contrast-enhanced computed tomography for predicting the activity and prognosis of acute pancreatitis

**DOI:** 10.1186/s13244-024-01738-0

**Published:** 2024-06-21

**Authors:** Ning Jun Yu, Xing Hui Li, Chao Liu, Chao Chen, Wen Han Xu, Chao Chen, Yong Chen, Ting Ting Liu, Tian Wu Chen, Xiao Ming Zhang

**Affiliations:** 1 Medical Imaging Key Laboratory of Sichuan Province, Department of Radiology, Affiliated Hospital of North Sichuan Medical College, No.1 South Maoyuan Road, Nanchong, 637001 Sichuan China; 2 Department of Radiology, The Second Clinical Medical College of North Sichuan Medical College Nanchong Central Hospital, Nanchong, Sichuan China; 3 Department of Radiology, Ruijin Hospital, Shanghai Jiao Tong University School of Medicine, Shanghai, China

**Keywords:** Radiomics, Computed tomography, Acute pancreatitis, Disease activity, The modified pancreatitis activity scoring system

## Abstract

**Background:**

The modified pancreatitis activity scoring system (mPASS) was proposed to assess the activity of acute pancreatitis (AP) while it doesn’t include indicators that directly reflect pathophysiology processes and imaging characteristics.

**Objectives:**

To determine the threshold of admission mPASS and investigate radiomics and laboratory parameters to construct a model to predict the activity of AP.

**Methods:**

AP inpatients at institution 1 were randomly divided into training and validation groups based on a 5:5 ratio. AP inpatients at Institution 2 were served as test group. The cutoff value of admission mPASS scores in predicting severe AP was selected to divide patients into high and low level of disease activity group. LASSO was used in screening features. Multivariable logistic regression was used to develop radiomics model. Meaningful laboratory parameters were used to construct combined model.

**Results:**

There were 234 (48 years ± 10, 155 men) and 101 (48 years ± 11, 69 men) patients in two institutions. The threshold of admission mPASS score was 112.5 in severe AP prediction. The AUC of the radiomics model was 0.79, 0.72, and 0.76 and that of the combined model incorporating rad-score and white blood cell were 0.84, 0.77, and 0.80 in three groups for activity prediction. The AUC of the combined model in predicting disease without remission was 0.74.

**Conclusions:**

The threshold of admission mPASS was 112.5 in predicting severe AP. The model based on CECT radiomics has the ability to predict AP activity. Its ability to predict disease without remission is comparable to mPASS.

**Critical relevance statement:**

This work is the first attempt to assess the activity of acute pancreatitis using contrast-enhanced CT radiomics and laboratory parameters. The model provides a new method to predict the activity and prognosis of AP, which could contribute to further management.

**Key Points:**

- Radiomics features and laboratory parameters are associated with the activity of acute pancreatitis.
- The combined model provides a new method to predict the activity and prognosis of AP.
- The ability of the combined model is comparable to the modified Pancreatitis Activity Scoring System.

**Graphical Abstract:**

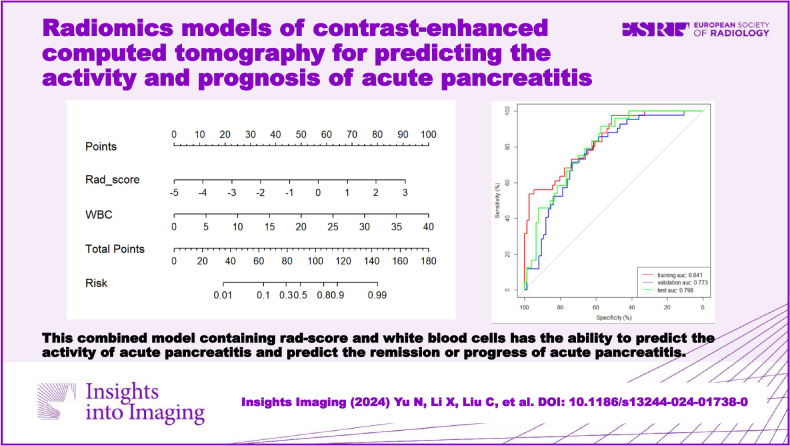

## Introduction

The incidence of acute pancreatitis (AP) has increased steadily in most Western countries [1]. AP has a highly variable course, and approximately 20% of patients develop to moderate or severe AP [2]. Severity of AP is a specific clinical outcome. Many clinical and imaging scoring systems for severity evaluation have been proposed. The activity of AP was defined as reversible manifestations during the course [3] and there are only some clinical scoring systems. Based on the status, this study intends to establish a new method for predicting the activity of AP.

The Pancreatitis Activity Scoring System (PASS) has been used to identify the condition of AP patients at any time and assess the response to current management. The five components of PASS include organ failure, SIRS, abdominal pain, morphine equivalent dose, and tolerance of a solid diet [3]. PASS is associated with important clinical outcomes [4], infectious pancreatic necrosis [5], etiologies [6] and courses [7]. Since the morphine equivalent dose is controversial, a modified Pancreatitis Activity Scoring System (mPASS) has been proposed [8]. However, mPASS does not include indicators that directly reflect pathophysiology processes and pancreatic situations on imaging.

According to 2012 RAC [9] and 2019 ACR [10], contrast-enhanced computed tomography (CECT) is important when the etiology is unknown, symptoms and signs are atypical on admission, and clinical condition is serious. Radiomics features could be more effective in reflecting the quantitative information acquired by images than those acquired by the naked eye [11]. It has been reported that radiomics is useful for predicting severity [12], peripancreatic necrosis [13], and recurrence of AP [11].

Radiomics can be used to obtain the situation of pancreatic parenchyma, and laboratory parameters are useful for directly reflecting the inflammatory processes of AP. The aims of the present study were as follows: (1) to determine the specific threshold of the mPASS score at admission in predicting severe AP; (2) to investigate the CECT radiomics features and the laboratory parameters of AP with a high and low level of disease activity; and (3) to build a nomogram based on the combined classification model to predict the activity and prognosis of AP.

## Materials and methods

### Patients

Inpatients with AP from January 2019 to October 2021 at institution 1 and from January 2021 to July 2022 at institution 2 (approved by the Ethics Committee, number 2021ER165-1, and obtained chictr220057403 of China Clinical Trial Registry) were consecutively enrolled in the present study, and individual consent for this retrospective analysis was waived.

Two or more of the following parameters were required to define AP: characteristic abdominal pain; serum amylase and/or lipase levels three or more times the upper limit of normal (35–135 U/L); and/or typical imaging findings of AP [9].

The inclusion criteria were as follows: (1) patients older than 18 years of age hospitalized for AP; (2) patients who were examined by two-phase CECT within 24 h of hospitalization; and (3) clinical data and laboratory parameters were completely collected at admission.

The exclusion criteria were as follows: (1) patients with cancer or severe chronic wasting disease; (2) patients with chronic pancreatitis; (3) unsatisfactory images or medical records; and (4) loss to follow-up such as a transfer to another hospital.

Patients who were diagnosed with AP were sought in the Hospital information system and Picture archiving and communication system. The patients from institution 1 were randomly segmented into training and validation groups at a ratio of 5:5. The patients from Institution 2 were served as the test group.

### CT technology and interpretation

Patients were scanned by a Somatom Definition AS + 128 (Siemens Healthineers) or LightSpeed VCT 128 (GE Healthcare, Boston) with intravenous administration of iodinated contrast material (Omnipaque, GE Healthcare). The details are provided in Supplementary Table 1 and S1.

Two radiologists (with 3 years of experience in pancreatitis imaging) evaluated CT severity index (CTSI) [14], extrapancreatic inflammation on CT (EPIC) scores [15], and local complications [9]. All findings were assessed by a consensus.

### Clinical parameters

Authors who did not know the imaging findings recorded the following parameters: age; sex; etiology; admission parameters related to AP [16–18], including white blood cell (WBC) count, platelet (Plt) count, Hematocrit (HCT), C-reactive protein (CRP), albumin (ALB), blood urea nitrogen (BUN), creatinine (Cr), lactic acid (Lac), total bilirubin (TBil) and calcium (Ca); mPASS score; acute physiology and chronic health evaluation II (APACHE II) score [19]; bedside index of severity in acute pancreatitis (BISAP) score [20]; severity of AP based on the modified Marshall score [9]; and length of hospital stay.

### Selection of the mPASS threshold for predicting severe AP

Mild AP patients without organ failure tended to demonstrate, on average, a low level of disease activity or experience a rapid decline in activity scores while severe AP patients with persistent organ failure demonstrated on average a high level of disease activity throughout the early course of illness [3]. ROC curves for predicting severe AP were drawn with severity as the state variable and admission mPASS as the test variable according to a previous method [4]. The cutoff value was selected as the threshold to divide patients into high and low level of disease activity group. Clinical outcomes and subsequent imaging findings were used to verify the accuracy of the threshold.

### Image segmentation and feature calculation

Image segmentation and feature calculation were performed in 3D Slicer (https://www.slicer.org). The radiologist outlined the pancreatic parenchyma on each slice of the arterial and venous phase CECT images, avoiding the bile duct and peripheral blood vessels. The radiomics features of sketched region of interest were extracted by a module called radiomics in 3D Slicer. Moreover, the features of original image were transformed by Gaussian and wavelet transforms. In addition, two radiologists outlined ROIs on 50 CT images for consistency testing.

### Preprocessing and consistency tests

The preprocessing steps included resampling and *Z* score normalization. Intraclass and interclass group correlation coefficients (ICCs) were analyzed to identify the consistency of the observers. ICC < 0.75 was considered to be of poor agreement.

### Dimensionality reduction feature screening

We performed the Mann‒Whitney *U* test or independent sample *t* test for features of the training group. Least absolute shrinkage and selection operator (LASSO) was used for dimension reduction. The model complexity was adjusted by changing the regularization parameter (λ) and optimized by 10 rounds of cross-validation.

### Model building and verification

Multivariable logistic regression was used to establish the radiomics model. The rad-score was calculated based on the radiomics formula and used to construct the combined model with meaningful laboratory parameters based on Institution 1.

### Construction and evaluation of the nomogram

The combined model was visualized as a nomogram. The calibration curve and decision curve were drawn. Because abdominal complications occur predominantly between the second and fifth weeks after episodes of AP [21], the prognosis was determined by subsequent imaging performed on CT/MRI and clinical manifestations during the period (Supplementary S3).

### Statistical analysis

Statistical analysis was performed using Statistical Package for Social Sciences (SPSS; Version 26.0, IBM, Armonk, NY, USA), and radiomics features were analyzed by R (v.4.2.1, https://www.r.project.org/). Independent *t* tests or Mann‒Whitney *U* tests were used for quantitative variable. Categorical variables were compared by χ2 test or Fisher’s exact tests. The test level was α = 0.05. The Glmnet package was used for LASSO regression, and the pROC package was used to generate ROCs. The rms and rmda packages were used for the nomogram and decision curve.

## Results

### Patients and threshold of mPASS in predicting severe AP

**Fig. 1:**
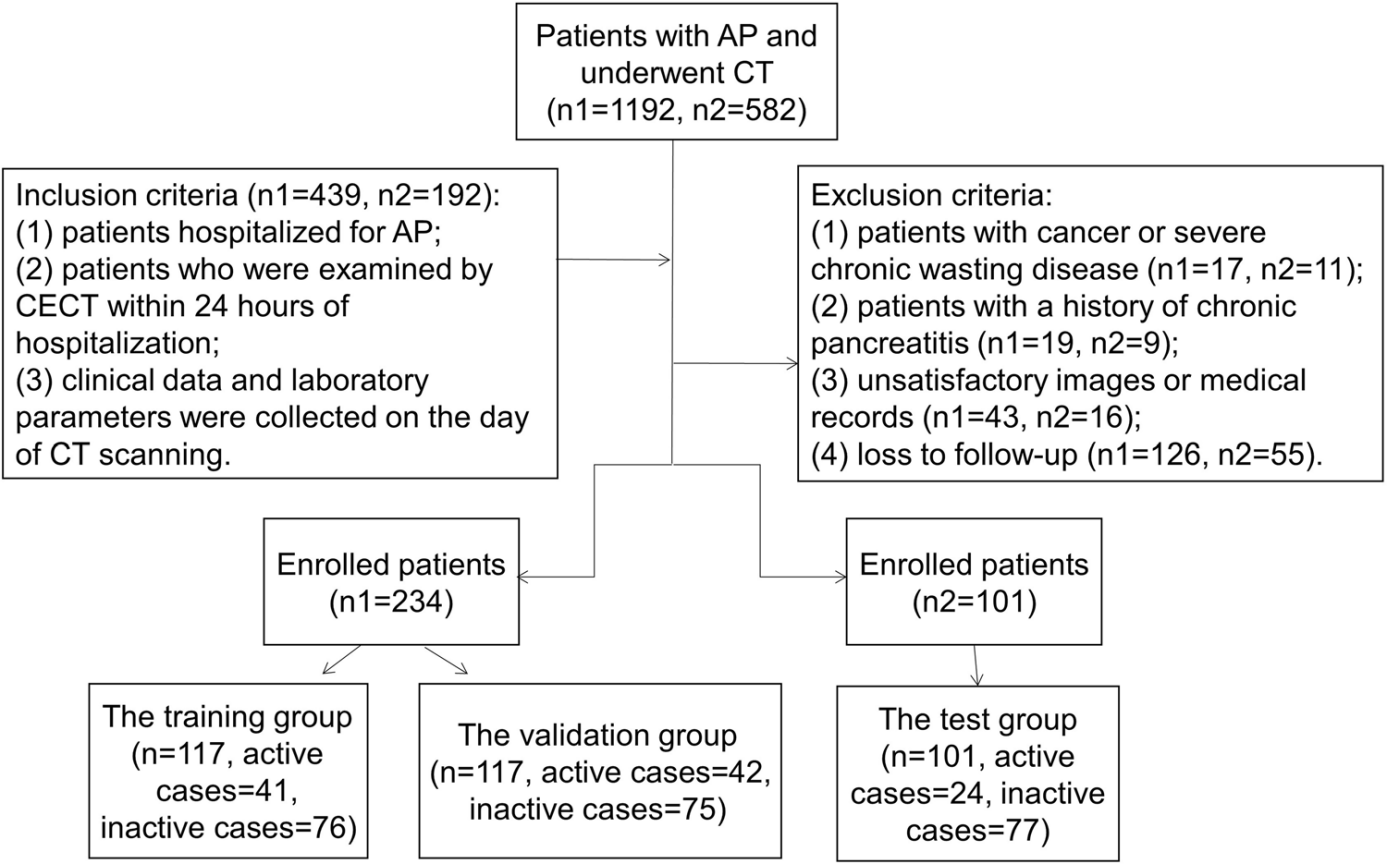
Flowchart of patients enrolled in the two institutions

The final study cohort consisted of 234 patients from institution 1 and 101 patients from institution 2. The study flowchart is shown in Fig. 1. The clinical and imaging characteristics are recorded in Table 1. For institution 1, 155 patients were males (66%), and the median age was 48 (IQR 38–58) years. The mean time from onset to admission was 4 days. The primary causes of AP were gallstones and hyperlipidemia, both accounting for 35% (82/234). Among the 234 patients, 2 patients died. For institution 2, 69 patients were male (68%), and the median age was 48 (IQR 39–61) years. The most common etiology was gallstones (39%). Among the 101 patients, 1 patient died. The median admission mPASS score was 75 (IQR 55–135) and 70 (IQR 55–110) in institutions 1 and 2 respectively.

**Table 1:** Clinical and imaging characteristics of the patients in the three groups

	Training group (*n* = 117)			Validation group (*n* = 117)			Test group (*n* = 101)		
	High level of disease activity (*n* = 41)	Low level of disease activity (*n* = 76)	*p*	High level of disease activity (*n* = 42)	Low level of disease activity (*n* = 75)	*p*	High level of disease activity (*n* = 24)	Low level of disease activity (*n* = 77)	*p*
Sex, *n* (%)			0.25			0.62			0.09
male	22 (54)	49 (65)		29 (69)	55 (73)		13 (54)	56 (73)	
female	19 (46)	27 (35)		13 (31)	20 (27)		11 (46)	21 (27)	
Age, median (IQR), y	49 (35.5–63)	51 (39–59)	0.6	48 (40–55)	45 (38–55)	0.44	53 (40–64)	47 (39–57)	0.21
Etiology, *n* (%)			0.86			0.55			0.19
Biliary	15 (37)	33 (43)		10 (23)	24 (32)		12 (50)	27 (35)	
Alcohol abuse	3 (7)	4 (5)		3 (7)	4 (5)		0 (0)	6 (8)	
Hyperlipidemia	12 (29)	20 (26)		17 (41)	33 (44)		8 (33)	19 (25)	
Other	11 (27)	19 (25)		12 (29)	14 (19)		4 (17)	25 (33)	
Severity, *n* (%)			< 0.001*			< 0.001*			< 0.001*
Mild	5 (12)	44 (58)		5 (12)	44 (59)		7 (29)	50 (65)	
Moderate severe	30 (73)	32 (42)		25 (60)	30 (40)		12 (50)	27 (35)	
severe	6 (15)	0 (0)		12 (29)	1 (1)		5 (21)	0 (0)	
Length of hospital stay, median (IQR), d	14 (9–16)	11 (7–14)	0.048*	13 (8–19)	9 (6–12)	0.003*	11 (6–18)	9 (7–11)	0.03*
APACHE II score, median (IQR)	7 (5–10)	4 (2–7)	< 0.001*	7 (5–10)	4 (2–7)	<0.001*	7 (4–8)	4 (2–5)	0.001*
BISAP score, median (IQR)	2 (1–2)	2 (1–2)	< 0.001*	1 (1–2)	0 (0–1)	<0.001*	2 (2–3)	1 (0–1)	< 0.001*
CTSI, median (IQR)	4 (4–6)	3 (2–4)	< 0.001*	5 (4–6)	3 (2–4)	<0.001*	4 (4–6)	3 (2–4)	0.001*
EPIC, median (IQR)	5 (4–7)	4 (1–5)	0.001*	5 (5–7)	3 (1–5)	<0.001*	6 (5–7)	4 (2–5)	< 0.001*
Local complication, *n* (%)	29 (71)	30 (40)	0.001*	31 (74)	31 (41)	0.001*	15 (63)	24 (31)	0.006*
Necrotizing, *n* (%)	15 (37)	10 (13)	0.008	24 (57)	7 (9)	<0.001	15 (63)	20 (26)	0.001

*APACHE II* acute physiology and chronic health evaluation II, *BISAP* bedside index of severity in acute pancreatitis, *CTSI* CT severity index, *EPIC* extrapancreatic inflammation on CT

* p value < 0.05

The cutoff value of admission mPASS in predicting severe AP was 112.5 in institution 1, and the AUC was 0.90 (95% CI: 0.83–0.96). The patients were divided into high or low level of disease activity group according to the threshold. In the training, validation and test group, there were 41, 42, and 24 cases with high levels of disease activity, and 76, 75, and 77 cases with low level of disease activity, respectively. There was no significant difference in the proportion of AP with high activity level among the three groups (35%, 36% and 24% in the training, validation and test groups, χ^2^ = 4.468, *p* = 0.11).

There was no significant difference in sex, age, or etiology between the high and low level of disease activity patients among the three groups (*p* > 0.05). The APACHE II, BISAP, CTSI, and EPIC scores, the length of stay, severity and local complication rate of high level of disease activity AP patients were higher than those of low level of disease activity AP patients in the three groups (*p* < 0.05). The AUC values for CTSI and EPIC in predicting activity of AP were 0.71, 0.77, 0.72 and 0.68, 0.71, 0.77 in the training, validation, and test group, respectively. WBC, ALB, and CRP levels of the AP patients with high activity level were higher than those of the AP patients with low activity level at institution 1 (*p* < 0.01). WBC count was an independent risk factor for predicting the activity of AP (OR = 1.075, 95% CI: 1.013–1.141, *p* = 0.017) (Table 2).

**Table 2:** Univariate and multivariate analyses in the main cohort

	All patients	High level of disease activity	Low level of disease activity	Univariate analysis	Multivariate	
	(*n* = 234)	(*n* = 83)	(*n* = 151)		OR (95% CI)	*p*
WBC, median (IQR), 10E9/L	12.42 (8.87–15.48)	13.18 (10.21–16.64)	11.95 (8.50–15.09)	< 0.001*	1.075 (1.013–1.141)	0.017*
PLT, median (IQR), 10E9/L	184 (142–217)	179 (136–224)	188 (144–216)	0.94		
HCT, mean (SD)	0.42 (0.07)	0.41 (0.08)	0.41 (0.07)	0.138		
ALB, median (IQR), g/L	40.90 (36.38–44.50)	40.70 (35.50–43.60)	41.20 (36.80–44.60)	0.006*		
Cr, median (IQR), µmol/L	63 (52–77)	65 (52–80)	61 (52–75)	0.678		
Lac, median (IQR), mmol/L	2.30 (1.71–3.07)	2.40 (1.71–3.20)	2.30 (1.70–3.06)	0.176		
TBIL, median (IQR), µmol/L	17.4 (12.1–26.2)	17.3 (12.6–26.2)	17.4 (11.6–26.8)	0.237		
BUN, median (IQR), mg/dL	13.36 (10.08–16.65)	14.67 (9.69–18.56)	13.08 (10.42–16.07)	0.282		
Hs-CRP, median (IQR), mg/L	68.27 (8.69–127.04)	83.91 (36.45–140.80)	34.19 (5.15–115.7)	< 0.001*		
Calcium, median (IQR), mmol/L	2.32 (2.19–2.41)	2.30 (2.15–2.41)	2.32 (2.21–2.41)	0.317		
Rad-score, median (IQR)	−0.79 (−1.77–0.25)	−0.18 (−0.84–0.86)	–1.16 (−2.25 to −0.38)	< 0.001*	2.063 (1.602–2.668)	< 0.001*

*WBC* white blood cell count, *Plt* platelet count, *HCT* red blood cell specific volume, *ALB* albumin, *Cr* creatinine, *Lac* lactic acid, *TBil* total bilirubin, *BUN* blood urea nitrogen, *CRP* C-reactive protein, *Ca* calcium

* *p* value < 0.05

### Feature calculation and consistency tests

Seven groups of radiomics features containing 1223 features were extracted (Supplementary S2). The satisfactory consistency rate of features of intraobserver and interobserver consistency reached 71% (median ICC = 0.93, IQR: 0.68–0.98) and 69% (median ICC = 0.92, IQR: 0.68–0.98) in the arterial phase, and 93% (median ICC = 0.98, IQR: 0.92–0.99) and 81% (median ICC = 0.96, IQR: 0.83–0.99) in the venous phase (Supplementary Fig. 1), respectively. Finally, 431 and 250 features in the arterial and venous phases respectively were removed.

### Dimensionality reduction feature screening

There were 36 and 17 features that showed significant differences (*p* < 0.05) tested by independent sample *t* test in the arterial and venous phases, respectively. The Mann‒Whitney *U* test showed that 191 and 86 features had significant differences, respectively. There were 227 and 103 features in the arterial and venous phases, respectively, submitted to LASSO regression. LASSO outputted 7 and 5 features with non-zero coefficients from the arterial and venous phase CECT for modeling respectively (Supplementary Fig. 2).

### Model building and verification

The AUC values of the radiomics model in the training, validation and test group were 0.79 (95% CI: 0.70–0.87), 0.72 (95% CI: 0.63–0.81) and 0.76 (95% CI: 0.65–0.87) in the arterial phase and 0.75 (95% CI: 0.67–0.84), 0.70 (95% CI: 0.61–0.80) and 0.72 (95% CI: 0.59–0.84) in the venous phase, respectively (Table 3).

**Table 3:** Performance of the radiomics model in the training, validation and test groups

Group	Phase	AUC	Accuracy	Sensitivity	Specificity	PPV	NPV
Training group	Arterial	0.79	74%	51%	86%	66%	77%
	Venous	0.75	67%	39%	82%	53%	71%
Validation group	Arterial	0.72	65%	33%	83%	52%	69%
	Venous	0.70	66%	36%	83%	54%	70%
Test group	Arterial	0.76	73%	46%	82%	44%	83%
	Venous	0.72	73%	58%	78%	45%	86%

*PPV* positive predictive value, *NPV* negative predictive value

**Fig. 2:**
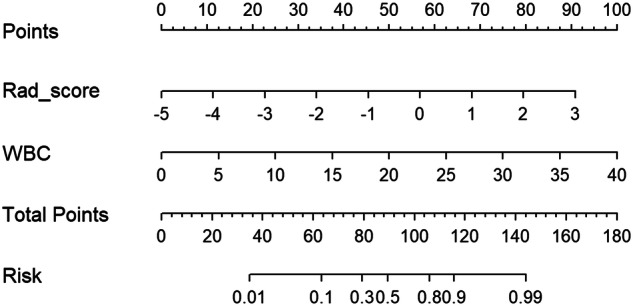
The nomogram incorporating WBC and rad-score for predicting AP activity risk was constructed in the training group

**Fig. 3:**
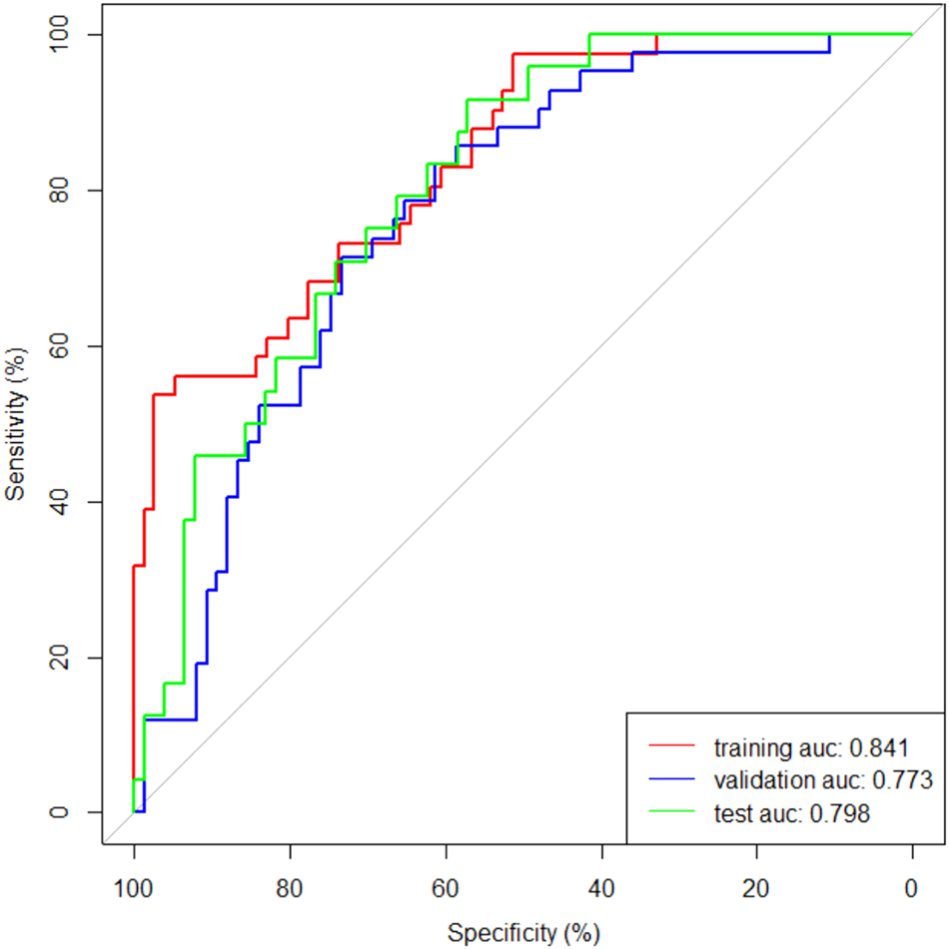
ROC curves of the combined model in the training, validation and test group

The final radiomics model was based on the optimal radiomics features of the arterial phase (Supplementary Fig. 3). The rad-score was calculated based on the radiomics formula (Supplementary Table 2). The WBC count with significance in the multivariate analysis and rad-score were contained to build the combined model and nomogram (Fig. 2). The AUC values for the combined model in predicting activity of AP were 0.84, 0.77 and 0.80 in the training, validation and test group, respectively (Fig. 3). The calibration curve demonstrated good consistency between prediction and observation (Supplementary Fig. 4). Decision curve analysis (DCA) showed that the use of the combined model helped patients obtain the greatest efficacy (Supplementary Fig. 5).

### Follow-up results

**Fig. 4:**
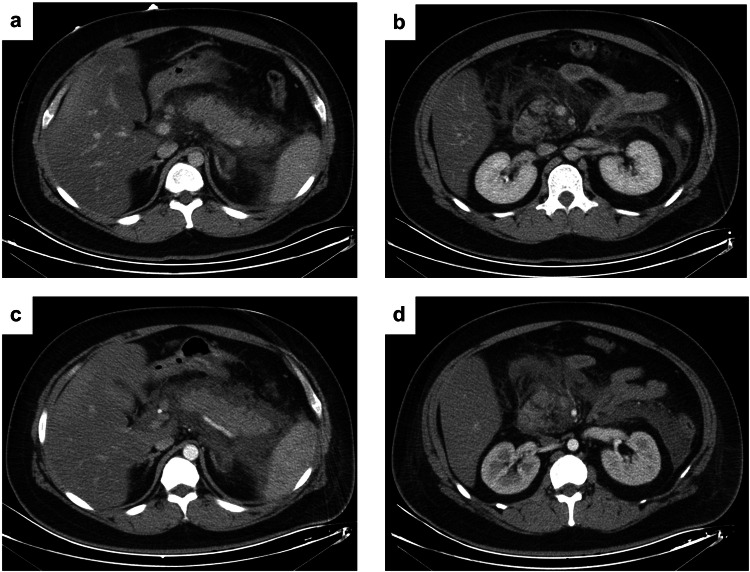
Axial CECT images of a 25-year-old man with AP whose admission mPASS score was 130. **a**, **b** The risk of high level of disease activity at admission was more than 80% based on the nomogram. **c**, **d** Acute peripancreatic fluid collection was increased after 7 days. Laboratory examination showed persistent respiratory failure and an elevated WBC count

**Fig. 5:**
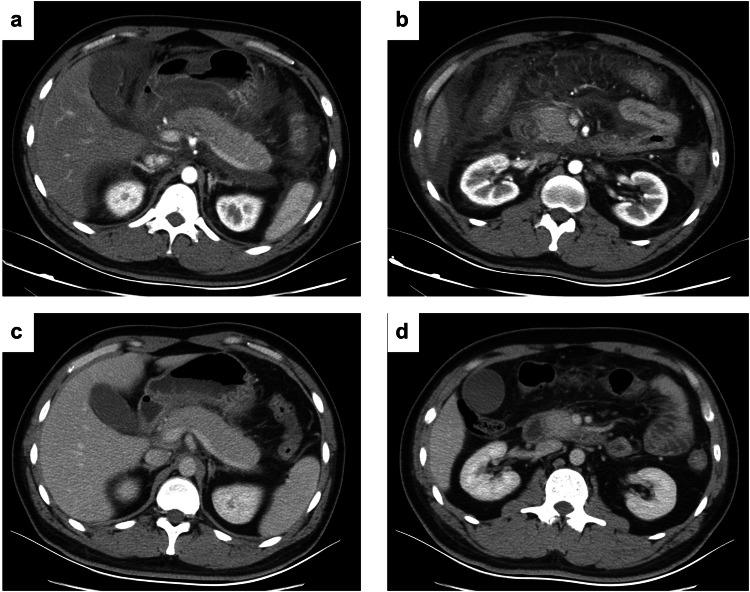
Axial CECT images of a 33-year-old man whose admission mPASS score was 105. **a**, **b** Acute peripancreatic fluid collection was observed at admission, and the risk of high level of disease activity was less than 50% based on the nomogram. **c**, **d** Acute peripancreatic fluid collection disappeared after 8 days

In the present study, all patients were followed up. During hospitalization or follow-up after discharge, the number of AP patients with a high level of disease activity whose second imaging findings, subsequent laboratory examinations, or symptoms showed no alleviation was 43 (43/107, 39%), and the proportion was higher than that in AP patients with low level of disease activity (20/228, 9%, χ2 = 47.068, *p* < 0.001) (Figs. 4 and 5). The AUC of the combined model in predicting the subsequent imaging findings or clinical manifestations with no alleviation was 0.76 (95% CI: 0.69–0.83), and that of the admission mPASS score was 0.77 (95% CI: 0.70–0.83).

## Discussion

In this double-center retrospective study, we determined the threshold of 112.5 of admission mPASS in predicting severe AP and constructed a combined model based on CECT radiomics and WBC, providing a new simple method for predicting the activity of AP. The AUC values of the combined model for predicting the activity of AP were 0.84, 0.77, and 0.80 in the training, validation, and test group, respectively. This study is the first attempt to predict the activity of AP using CECT radiomics and laboratory parameters.

The AUC of admission mPASS scores in predicting severe AP was 0.90 at institution 1, which was similar to a previous study [8]. In the present study, the length of stay, severity, and complication rate of AP patients with high level of disease activity at admission were higher than those of AP patients with low level of disease activity, which showed no difference with previous studies [4]. The follow-up results showed that patients with high level of disease activity at admission were more likely to show imaging findings and clinical manifestations with no alleviation. These results verified the accuracy of mPASS > 112.5 at admission in predicting important clinical outcomes and imaging with no remission.

In the present study, significant differences were found in the APACHE II and BISAP between the high and low level of disease activity groups, which may be because of the overlapping components of severity score and mPASS, such as organ failure and SIRS. WBC count was the only independent risk factor to predict the activity of AP. Previous research has shown that trypsin activation and neutrophil infiltration are mutually reinforcing [22], and toxic mediators can cause tissue injury [23]. Although ALB and CRP levels are associated with severe AP [18, 24], additional research is required to judge whether ALB and CRP can be used for predicting the activity of AP.

The definition of disease activity is reversible manifestations during the course while severity is a fixed state or outcome [3]. In the present study, there were significant differences in the CTSI and EPIC scores between the high and low level of disease activity groups, which suggested that imaging may be able to identify an association between the severity and activity. However, conventional imaging performance often lags behind disease progression. Minimal activity changes may cause severe fluctuations in radiomics scores, while conventional imaging scoring systems may change a little or not at all.

Radiomics plays a vital role in the study of AP [11–13]. Recently, Zhao Y et al [25] emphasized the application of radiomics in AP severity, but studies have not reported whether radiomics can be used to predict AP activity. In the present study, the CECT radiomics features showed the ability to predict the activity of AP. Compared to CTSI and EPIC, radiomics model had a superior value. It suggested that radiomics features revealed some of the differences between high and low level of disease activity, which was possibly due to some small morphological changes in pancreatic parenchyma caused by hemoconcentration, decreased blood flow, decreased tissue oxygenation decreased, vasoconstriction or increased permeability [26, 27].

The effectiveness of the radiomics model was improved by incorporating WBC count. The compositions of the combined model were easily acquired from CECT and routine blood tests, thus avoiding additional medical tests. The follow-up results showed that patients with high total points based on the nomogram were more likely to have a high risk of high activity level at admission and show an increasing trend in imaging and clinical performance, which may be associated with the development of irreversible liquefaction and necrosis from transient ischemia and hypoxia of pancreatic parenchyma [28]. The follow-up results showed that the ability of the combined model in predicting the clinical and imaging with no remission is comparable to the admission mPASS, and confirmed the potential clinical application value of the combined model as a quantitative instrument in predicting the activity and prognosis of AP.

The model in the present study showed a stable performance. The performance in the test group from another tertiary referral center showed that the model had a certain generalization ability. Resampling is used to diminish the unsatisfactory reproducibility of radiomics features. The radiomics features are linearly separable, and multiple logistic regression has a good stability, direct calculation process, and good performance.

There were several limitations in the present study. First, more clinical characteristics and laboratory parameters must be considered in a comprehensive evaluation. However, conventional laboratory parameters related to AP severity were used in the present study to avoid additional costs. Second, although 335 cases are sufficient for radiomics, more cases needed to be brought in to increase universality and credibility. Third, there may be some confounding factors and biases in this retrospective study. We developed specific criteria and used external validation to ensure the reliability of the model. Finally, the confounding role of pancreatic and peripancreatic fat necrosis in the underestimation of the disease severity that has not been assessed in this study.

In conclusion, mPASS score of 112.5 at admission is a meaningful threshold in predicting severe AP, and radiomics features may reflect the differences between AP with high and low level of disease activity, which are hidden in the pancreatic parenchyma. WBC may reflect the differences between inflammatory processes of AP with distinct activity. The combined model based on CECT radiomics provided a new method to predict the activity of AP, and had a potential value in predicting clinical or imaging with no remission. It is expected to be a promising tool for predicting the activity and prognosis of AP, which could contribute to further management.

### Supplementary information

ELECTRONIC SUPPLEMENTARY MATERIAL

## Data Availability

Data and materials are available at China Clinical Trial Registry.

## Abbreviations

AP: Acute pancreatitis
APACHE II: Acute physiology and chronic health evaluation II
AUC: Area under the receiver operating characteristic curve
BISAP: Bedside index of severity in acute pancreatitis
CECT: Contrast-enhanced computed tomography
CTSI: Computed tomography severity index
EPIC: Extrapancreatic inflammation on Computed tomography
ICC: Intraclass correlation coefficient
LASSO: Least absolute shrinkage and selection operator
mPASS: The modified Pancreatitis Activity Scoring System
PASS: The Pancreatitis Activity Scoring System
ROC: Receiver operating characteristic curve
WBC: White blood cell
