# Supplementary material for: Radiomics models of contrast-enhanced computed tomography for predicting the activity and prognosis of acute pancreatitis
Source: Insights Imaging. 2024 Jun 21;15:158. doi: 10.1186/s13244-024-01738-0 (PMC11190132; doi:10.1186/s13244-024-01738-0)
Supplement: Supplementary file 1 — ELECTRONIC SUPPLEMENTARY MATERIAL [file 13244_2024_1738_MOESM1_ESM.pdf]

Radiomics models of contrast-enhanced computed tomography for predicting the activity and prognosis of acute pancreatitis

ELECTRONIC SUPPLEMENTARY MATERIAL

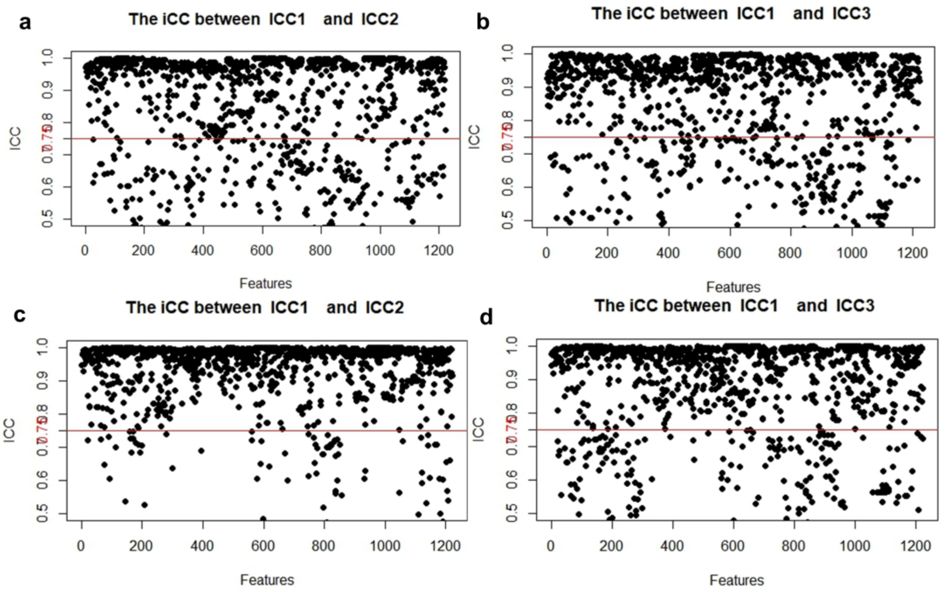

Supplementary Fig. 1 Interobserver and intraobserver agreement based on the interclass correlation coefficient (ICC). ICCs > 0.75 were considered to be consistent. (a) Intraobserver agreement in arterial phase images. (b) Interobserver agreement in arterial phase images. (c) Intraobserver agreement in venous phase images. (d) Interobserver agreement in venous phase images.

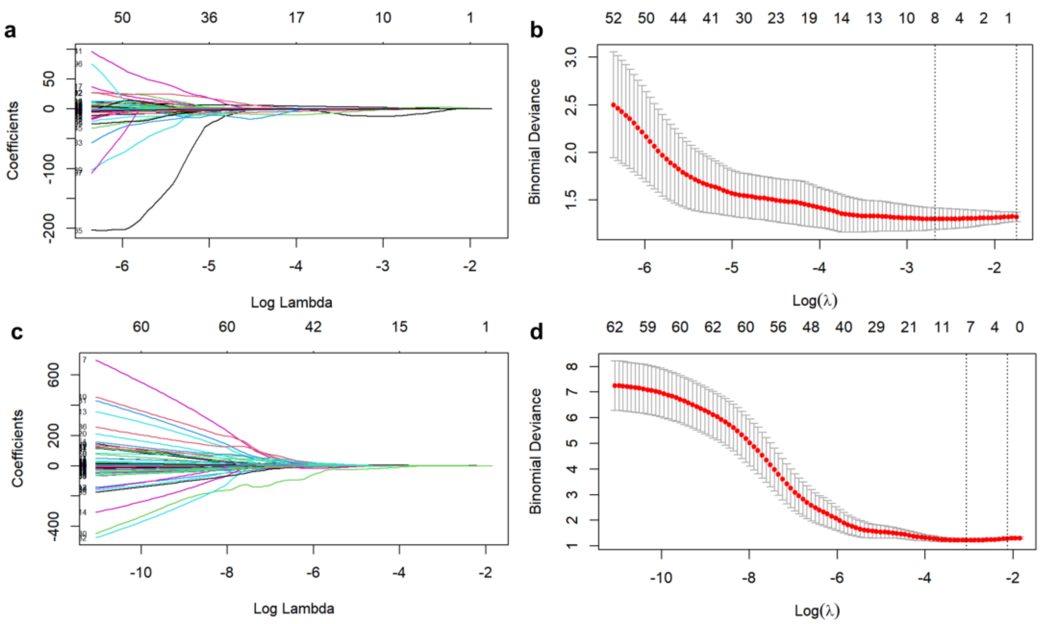

Supplementary Fig. 2 Least absolute shrinkage and selection operator (LASSO) regression was used to select features. (a, c) LASSO coefficient profiles for the 227 radiomics features in the arterial phase and 103 radiomics features in the venous phase. (b, d) Tuning parameters (λ) using 10-fold cross-validation via minimum criteria. The ends of the vertical line indicate the value of log (λ) and 7 features in the arterial phase and 5 features in the venous phase, which had nonzero coefficients.

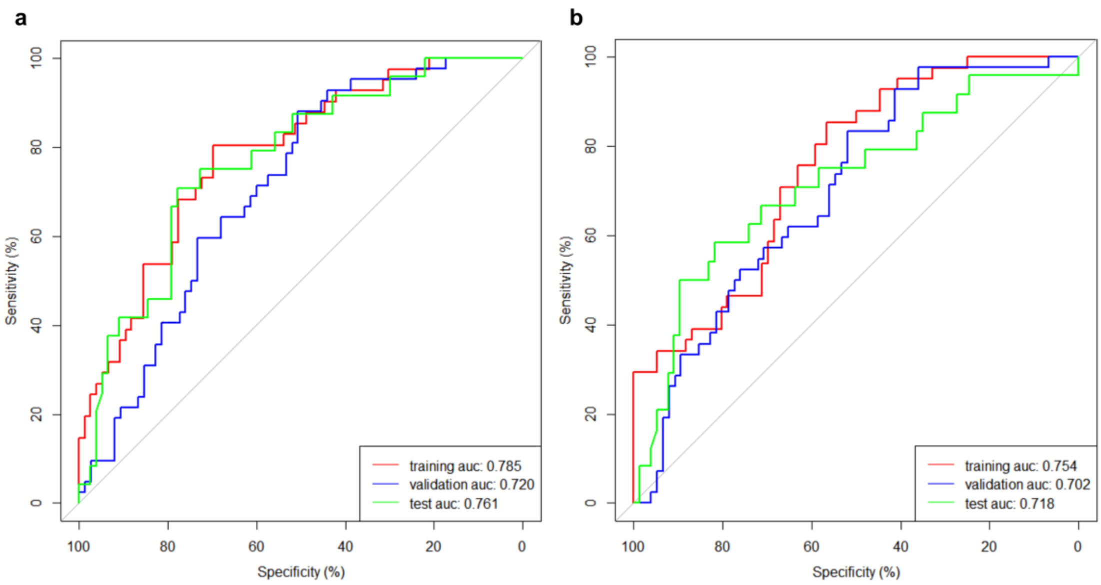

Supplementary Fig. 3 ROC curves of the radiomics model in the arterial phase (a) and venous phase (b) of the training group, validation group and test group.

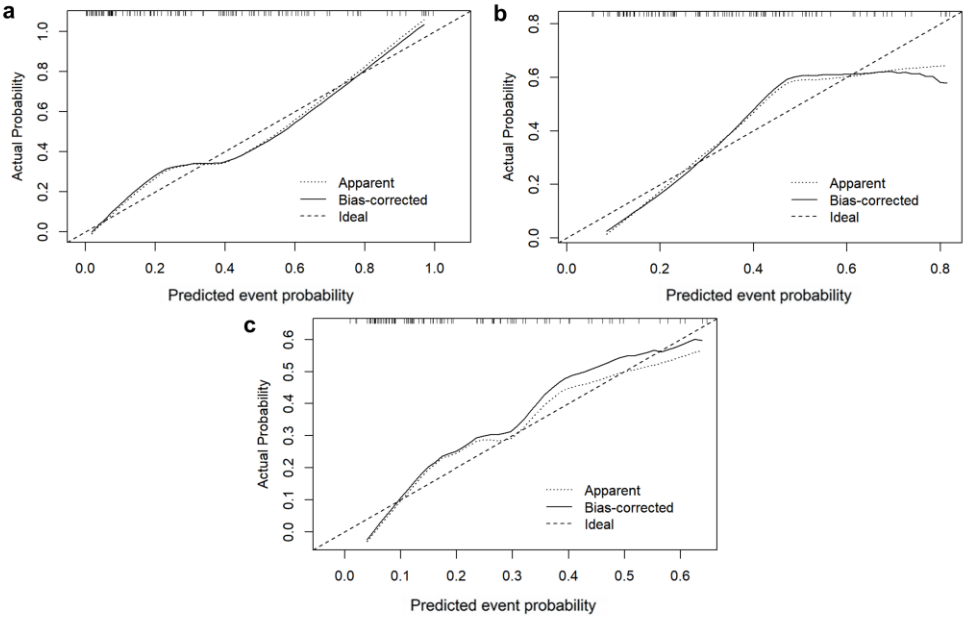

Supplementary Fig. 4 Calibration curves of the radiomics nomogram in the training group (a), validation group (b) and test group (c).

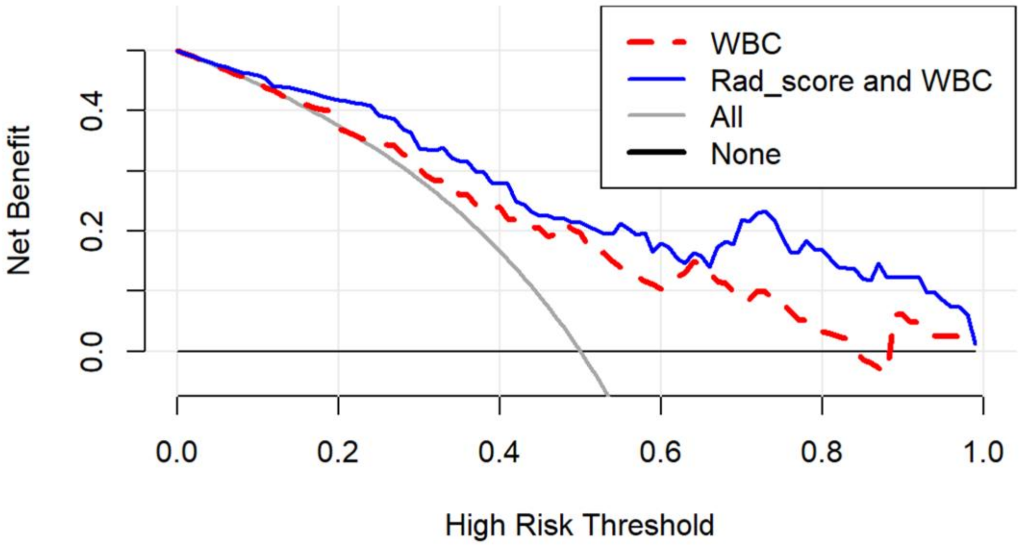

Supplementary Fig. 5 Decision curve analysis for the nomogram.

Supplementary Table 1 CT scan parameters

Scanning Machines	Tube Voltage, kV	Tube Current, mA	Matrix	FOV, cm	Reconstruction Kernel	Section Thickness, mm	Pitch	Collimation, mm
GE LightSpeed VCT	120	200	512×512	35×35	B30f	5	0.9	64×0.6
Somatom Definition AS+	120	200	512×512	35×35	B30f	5	1	128×0.6

Supplementary Table 2 Parameters and coefficients of the radiomics formula

Serial number	Parameters	Coefficients
	Intercept	30.3296
N5	original_shape_Maximum2DDiameterColumn	5.3384
N444	log-sigma-2-0-mm-3D_glrlm_GrayLevelNonUniformityNormalized	-3.3058
N103	original_ngtdm_Busyness	0.6589
N150	log-sigma-0-5-mm-3D_gldm_DependenceEntropy	-28.1031
N389	log-sigma-2-0-mm-3D_firstorder_Energy	-0.6047
N524	wavelet-LLH_gldm_DependenceNonUniformityNormalized	-4.0034
N1211	wavelet-LLL_glszm_SizeZoneNonUniformity	-1.2347

S1 CT image acquisition

After a routine non-enhanced scan, arterial and venous phase CECT were implemented after 35 – 40 s and 65 – 70 s delays following the intravenous administration of iodinated contrast material (Omnipaque, GE Healthcare) at 1.5 mL per kilogram at a rate of 3.5 – 4 mL/s using a pump injector (Ulrich CT Plus 150, Ulrich Medical).

S2 Seven groups of features

Seven groups of radiomics features were extracted, including first order features, shape features, gray-level cooccurrence matrix, gray-level run-length matrix, gray level size zone matrix, neighboring gray tone difference matrix and gray level dependence matrix.

S3 Definitions of follow-up imaging findings and clinical manifestations with no alleviation

Clinical manifestations with no alleviation after admission were described by the following symptoms: respiratory failure, SIRS, increased infection, recurrent abdominal pain and conditions requiring rescue.

Second imaging findings with no alleviation were described by the following symptoms: increased pancreatic swelling; new or expanded parenchymal or peripancreatic necrosis; increased ascites; and increased mesenteric inflammation.
